# Improved moderation for gene-wise variance estimation in RNA-Seq via the exploitation of external information

**DOI:** 10.1186/1471-2164-14-S1-S9

**Published:** 2013-01-21

**Authors:** Ellis Patrick, Michael Buckley, David Ming Lin, Yee Hwa Yang

**Affiliations:** 1 School of Mathematics and Statistics, University of Sydney, Sydney NSW 2006, Australia; 2 CSIRO Mathematical and Information Sciences, Private Bag 33, Clayton South 3168, Australia; 3 Department of Biomedical Sciences, Cornell University, Ithaca, NY, USA

## Abstract

**Background:**

The cost of RNA-Seq has been decreasing over the last few years. Despite this, experiments with four or less biological replicates are still quite common. Estimating the variances of gene expression estimates becomes both a challenging and interesting problem in these situations of low replication. However, with the wealth of microarray and other publicly available gene expression data readily accessible on public repositories, these sources of information can be leveraged to make improvements in variance estimation.

**Results:**

We have proposed a novel approach called Tshrink+ for inferring differential gene expression through improved modelling of the gene-wise variances. Existing methods share information between genes of similar average expression by shrinking, or moderating, the gene-wise variances to a fitted common variance. We have been able to achieve improved estimation of the common variance by using gene-wise sample variances from external experiments, as well as gene length.

**Conclusions:**

Using biological data we show that utilising additional external information can improve the modelling of the common variance and hence the calling of differentially expressed genes. These sources of additional information include gene length and gene-wise sample variances from other RNA-Seq and microarray datasets, of both related and seemingly unrelated tissue types. The results of this are promising, with our differential expression test, Tshrink+, performing favourably when compared to existing methods such as DESeq and edgeR when considering both gene ranking and sensitivity. These improved variance models could easily be implemented in both DESeq and edgeR and highlight the need for a database that offers a profile of gene variances over a range of tissue types and organisms.

## Background

In the post-genomic era, the development of technologies for sequencing the genome and transcriptome has become a key issue in the global analysis of biological systems. Even with the lowering cost of sequencing data, the majority of RNA-Seq experiments are still suffering from low replication numbers. The identification of differential expressed (DE) genes and transcripts is still a key question of interest in many biological studies. To date, there are many methods that provide a test of whether a gene is DE or not [1], including cufflinks [2], DESeq [3] and edgeR [4]. A feature in all of these methods is moderation of gene-wise variance estimates to improve DE inference. Moderation is important in small samples size comparisons, increasing both the power and accuracy of a DE test [5]. The key differences between these methods are the extent of the moderation and their *common variance *estimate--the variance estimate that the procedure is moderating towards.

DESeq and edgeR account for the heteroscedasticity observed in the read counts of genes by modelling the relationship between expected value of the count and its variability. We propose using additional information, such as gene length and variance estimates from external datasets, as explanatory variables to further model the heterogeneity seen in the observed gene variances. Combining these improved models of gene variance with a moderation method [6] creates a robust tool for estimating gene variances and hence calling differential gene expression. When evaluated on publicly available data this tool offers both improved gene ranking and power of detection when compared to DESeq and edgeR. This method is implemented in the R package sydSeq available on http://www.maths.usyd.edu.au/u/jeany/software.htm.

### RNA-Seq

The development of high throughput sequencing technologies has made it possible to sequence the transcriptome at a much higher resolution and coverage than was previously available. Sequencing of cDNA samples (RNA-Seq) has a dynamic range larger than that of microarrays [7]. This, combined with its high level of reproducibility [8] and falling cost, makes high throughput sequencing technologies an increasingly attractive alternative to microarrays for transcriptome analysis. In the following we will describe how variances are estimated for RNA-Seq data with small samples sizes.

A typical RNA-seq data analysis work flow consists of many steps. These steps generally consist of mapping, summarisation, normalisation, differential expression analysis and systems biology [9]. The summarisation step counts how many reads were mapped to each gene or transcript. We will consider the case of mapping to genes rather than transcripts, so for us summarisation results in a matrix of counts, where the rows and columns correspond to genes and samples respectively.

Let *y_ij _*be the observed read count for the *i^th ^*gene in the *j^th ^*sample where sample *j *belongs to treatment *t*(*j*) = 1, 2. Let $\sigma_{i}^{2}$ and *μ_i _*be the variance and mean read count for gene *i*. For ease of presentation we will assume that all effects that are generally normalised for or modelled, such as library sizes and GC content, remain constant across samples. The technical variability for a gene count in RNA-Seq can be modelled quite reliably as Poisson [10,11]. This is attractive in situations of low replication as one parameter can be estimated to describe both the mean and variance of a gene. Modelling the data as Poisson will give a very reliable estimate of which genes have changed in expression between any two samples. However many experiments are not simply focused on the detection of gene expression differences between any two samples focusing instead on the differences between any two types of cells for example. This distinction is important as it requires us to not only model the technical variability of the experiment but to also model the biological variability of a particular cell type (or experimental condition).

An over-dispersed Poisson, a discrete distribution with dispersion greater than a Poisson, can be modelled using a Negative Binomial. A negative binomial random variable, *Y *, can be parametrised as

(1) $$P\left(Y=y\right)=\left(\begin{array}{c} \begin{array}{c} r+y-1\\ y \end{array} \end{array}\right)p^{r}{\left(1-p\right)}^{y}.$$

This standard formulation is generally referred to as NB2. Under this formulation, the biological variability of the expression of a gene is modelled as a quadratic function of its mean expression *μ*:

(2) $$\sigma^{2}=\mu +b\mu^{2},$$

where as $b=\frac{1}{r}$ gets small the negative binomial will approach a Poisson. The parameter *b *has been referred to as the coefficient of biological variation. A negative binomial is generally parametrised as a function of *r *and *p*. However, by parametrising a negative binomial in terms of its mean *μ *and variance *σ*^2 ^where

(3) $$r=\frac{\mu^{2}}{\sigma^{2}-\mu }$$

(4) $$p=\frac{\mu }{\sigma^{2}}$$

and *σ*^2 ^>*μ*, a negative binomial can then be used to model counts that have untraditional mean-variance relationships. This relationship is generally expressed as

(5) $$\sigma^{2}=\mu +f\left(\mu \right)$$

where *f*(*μ*) explains the biological variability can be fitted by some form of local regression [3]. This formulation of *σ*^2 ^highlights that *σ*^2 ^should always be greater than or equal to *μ*.

In current RNA-Seq experiments it is still quite common to see experiments with very little biological replication. Estimating variances from few observations is unstable [12]. To improve the stability and accuracy of these variance estimates there have been many methods proposed to shrink the variances to some common value for microarrays [12] and RNA-Seq [1]. We will refer to this as moderation. By stabilising the variances and sharing information moderation also increase the power of a statistic as this increases the degrees of freedom of a variance estimate [5].

### Heterogeneous gene variances

It is well accepted that some genes have a higher variance than other genes [13]. That is, some genes vary in expression more from cell to cell, person to person, or treatment to treatment than other genes. In RNA-Seq datasets, genes with larger average expression have on average larger observed variances. Instead of shrinking the estimate of a genes variance towards some common value (as is often done in microarrays) to improve stability [12], edgeR and DESeq shrink the estimate towards some fitted curve describing the relationship between mean and variance. We refer to this fitted curve as the *common variance*. In doing this they are making the strong assumption (although not an unreasonable one) that genes with a similar average count should have a similar variance.

We incorporate external data from RNA-Seq and microarrays on mouse striatum and RNA-Seq data from different tissues to better estimate variances and hence identify DE genes between C57BL/6J and DBA/2J mouse striatum samples.

## Methods

### Tshrink+

We propose using local regression [14] to fit a smoothed surface through any number of variables (γ_(1)_, γ_(2) _...) that may help to explain the observed pooled sample variances ${{\hat{\sigma }}^{2}}_{gene}=s^{2}$. We estimate the common variances $\sigma_{common}^{2}$ as

(6) $${\hat{\sigma }}_{common}^{2}=\mu +f\left(\mu ,{\text{γ}}_{\left(1\right)},{\text{γ}}_{\left(2\right)},..\right).$$

When using variance estimates from other RNA-Seq experiments, these variances will also have a very strong mean-variance relationship. For use as an explanatory variable we normalise the external variance estimates in such a way that they have mean zero and variance one for all ranges of expression.

To illustrate how this improved common variance can aid in moderation we propose using a quasi-empirical Bayes moderation method [6], where the variance is moderated as

(7) $${\hat{\sigma }}_{shrink}^{2}=\lambda {\hat{\sigma }}_{common}^{2}+\left(1-\lambda \right){\hat{\sigma }}_{gene}^{2},$$

and ${\hat{\sigma }}_{shrink}^{2}$, ${\hat{\sigma }}_{common}^{2}$ and ${\hat{\sigma }}_{gene}^{2}$ are the moderated, common and sample variances. Without making distributional assumptions, the shrinkage parameter *λ *can be estimated by the equation

(8) $$\lambda =\min\;\left(1,\;\frac{\sum_{k=1}Var\;\left(\sigma_{k\left(gene\right)}^{2}/\sigma_{k\left(common\right)}^{2}\right)}{\sum_{k=1}{\left({\hat{\sigma }}_{k\left(gene\right)}^{2}/{\hat{\sigma }}_{k\left(common\right)}^{2}-1\right)}^{2}}\right).$$

*λ *is the ratio of the expected and average squared error of the common variance estimate. Due to the large amount of smoothing that is used in estimating the common variance, we will make the assumption that the data, standardised using the common variance estimate, is approximately standard normal. Under this assumption the variance of $\sigma_{k\left(gene\right)}^{2}/\sigma_{k\left(common\right)}^{2}$ which has *n *- 2 degrees of freedom is 2/(n - 2). Our estimate of *λ *then becomes

(9) $$\lambda =\min\;\left(1,\;\frac{2n}{\left(n-2\right)\sum_{k=1}{\left({\hat{\sigma }}_{k\left(gene\right)}^{2}/{\hat{\sigma }}_{k\left(common\right)}^{2}-1\right)}^{2}}\right).$$

A Wald test for each gene is then performed using the statistic

(10) $$\frac{{ȳ}_{t_{1}}-{ȳ}_{t_{2}}}{\sqrt{\sigma_{shrink}^{2}\;\left(\frac{1}{n_{1}}+\frac{1}{n_{2}}\right)}},$$

where we utilise the Welch-Satterthwaite equation [15,16] to estimate its degrees of freedom $\hat{v}$. We have assumed earlier that that the degrees of freedom corresponding to common variance is *ν_common _*= ∞ and can thus estimate *v*_k _as

(11) $${\hat{v}}_{k}=\frac{{\left(\lambda {\hat{\sigma }}_{k\left(common\right)}^{2}+\left(1-\lambda \right){\hat{\sigma }}_{k\left(gene\right)}^{2}\right)}^{2}}{\frac{{\left(1-\lambda \right)}^{2}}{v_{gene}}{\hat{\sigma }}_{k\left(gene\right)}^{4}}$$

where *ν_gene _*= *n *- 2. For simplicity, rather than using a different *v *for each gene we instead use one degrees of freedom estimate, *v_shrink_*, for all genes, taken as the mean of the ${\hat{v}}_{k}$'s.

### Data

#### Bottomly dataset

The Bottomly data [17] was used as the main analysis dataset for evaluation and was chosen because of its relatively large number of biological replicates. The pre-processed RNA-Seq data comparing ten C57BL/6J (B6) and eleven DBA/2J (D2) mouse striatum was downloaded from the ReCount project [18] as a matrix of counts. For simplicity only the first ten DBA/2J samples were used. All data used in the analysis are normalised counts as DESeq and edgeR do not accept gene-wise normalisation factors. To model the disparate library sizes and biases of PCR amplification observed in the data, a cyclic robust linear model was used. Using the first sample in the dataset as a reference, M values were calculated for each gene in the remaining samples and a straight line was fitted through the M-values using GC-content as an explanatory variable. The M-values were then normalised to this line such that the average M-value was zero over the range of GC-content. After this normalisation there were still batch and other sample specific effects evident in the data. These were normalised out using a cyclic loess [19] strategy as described in Additional File 1. This normalisation appeared to be more suitable than RUV [20] and SVA [21] improving concordance with microarray results as seen in Additional Figures 1 and 2 in Additional File 1 .

**Figure 1:**
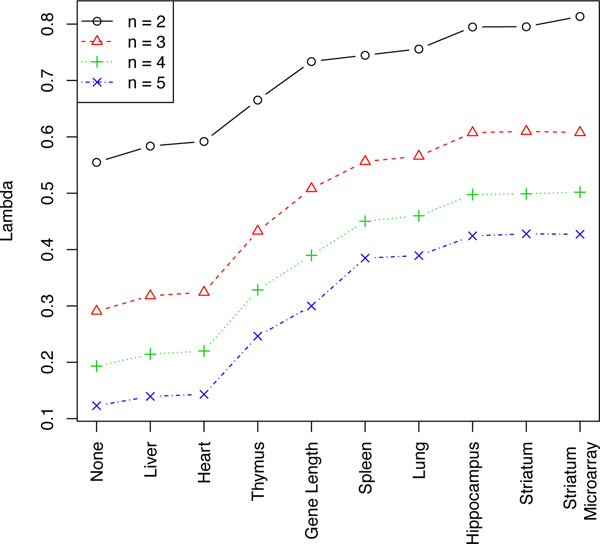
**Effect of utilising different sources of information on the estimation of *λ***. Variance estimates from the external datasets (Table 1) and gene length are used to aid in the estimation of the common variance functions of one hundred comparisons of *n *B6 and *n *D2 mouse striatum samples. The average *λ *value is plotted for each *n *comparison and information source for *n *ranging from two to five. *λ *is the ratio of the expected and average squared error of the gene sample variance to the common variance. The information source "None" corresponds to using no extra information, "Striatum" the RNA-Seq samples from Polymenidou et al (2011) and "Striatum Microaray" the microarray striatum samples from Bottomly et al (2011). The information sources have been sorted by their *λ *values for *n *equals two.

**Figure 2:**
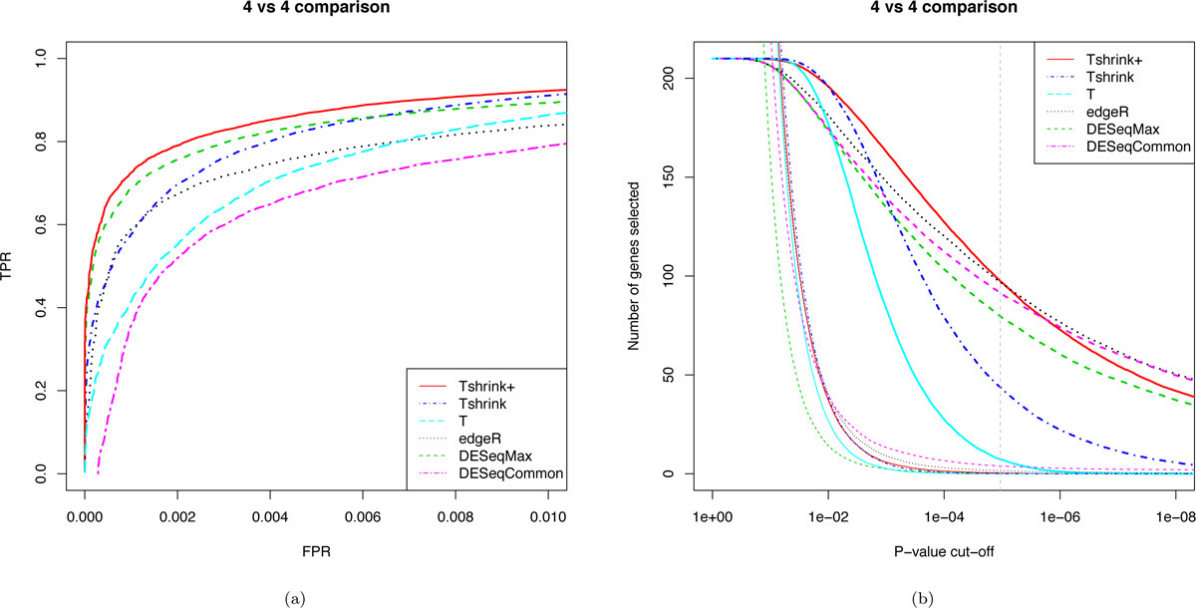
**Comparing six DE methods on a 4 vs 4 comparison**. One hundred random comparisons of four B6 and four D2 mouse striatum samples for six DE methods. Average TP and FP are calculated for the full range of p-value cut-offs. The TPR and FPR are plotted against each other in a) to form ROC curves and displayed in the region for FPR less than 0.01 as this is most relevant for calling DE. For any given FPR a method with a larger TPR is deemed to have ranked the genes better. In b) the number of TP (in bold) and FP are plotted for a range of p-value cut-offs. The x-axis is in log-scale. The grey dashed vertical line corresponds to a Bonferroni adjusted cut-off of 0.05.

#### External datasets

Sample variances from three datasets were used as sources of additional information to aid in the estimation of the common variance. These are described in Table 1. All RNA-Seq data were mapped to the mm9 mouse genome using bowtie [22] and normalised for GC content bias and library size differences as the Bottomly dataset was. The microarray data were read and processed using the R packages Affy [23] and gcrma [24].

**Table 1:** Additional information sources.

Species	Tissue	Replicates	Platform	Source	GEO accession
	Liver	6			
	Spleen	6			
C57BL/6J mouse	Thymus	6	RNA-Seq	Keane *et al. *(2011) [25]	GSE30617
	Lung	6			
	Heart	6			
	Hippocampus	6			

C57BL/6J mouse	Striatum	4	RNA-Seq	Polymenidou *et al.* (2011) [27]	GSE27218

C57BL/6J mouse	Striatum	10	microarray	Bottomly *et al.* (2011) [17]	GSE26024

Variance estimates from these three datasets are be used to improve the estimation of the common variance function in the main analysis dataset.

### Evaluation study

In this study, we evaluate our proposed method of improving variance estimation for differential gene expression analysis, Tshrink+. This evaluation consists of two components, assessing the capacity of a common variance estimate to explain the observed gene sample variances and evaluating how improving this common variance estimate can aid in the detection of differentially expressed genes. The performance of Tshrink+ will also be compared with two commonly used packages, edgeR and DESeq. This evaluation study is built upon one main dataset, the Bottomly data, and three datasets which are used for additional information.

In order to assess the capacity of a common variance estimate to explain the observed gene sample variances we will use the shrinkage coefficient *λ*, described in the methods section, as a statistic. *λ *is the ratio of the expected and average squared error of the common variance estimate. We aim to assess the effectiveness of using information in addition to the average expression of a gene to estimate a common variance function. Variance estimates from the external datasets described in Table 1 and also gene length are used to aid in the estimation of the common variance functions of one hundred random comparisons of *n *samples of B6 mouse striatum tissue with *n *samples of D2 mouse striatum tissue. This is performed for one additional dataset at a time. The average *λ *value is calculated for each *n *comparison and information source using only the genes that are present in all data sources.

We then further demonstrate that improving the information content of an additional information source improves the estimation of the common variance. This will be achieved by using variance estimates from the D2 mice to aid in the estimation of a common variance function of the B6 mice. The variance estimates from a random *n *D2 mouse samples are used to estimate the common variance function of a random four B6 mouse, this is repeated one hundred times and average *λ *values are calculated.

We will assess the influence of using additional information and moderation on the detection of differentially expressed (DE) genes. To do this we compare

1. a t-test (T),

2. a moderated t-test (Tshrink) and

3. a moderated t-test using additional information (Tshrink+).

These will also be compared to

4. DESeq using only the common variance (DESeqCommon),

5. DESeq using the maximum of the common variance and sample variance (DESeqMax) and

6. edgeR using a trended common variance and empirical Bayes to shrink the gene sample variances towards the common variance (edgeR).

To assess the effectiveness of the six DE methods, a standard t-test was performed comparing ten B6 and ten D2 mouse striatum samples. In all of the following, the results of this t-test are taken to be the "truth". From this t-test a gene is conservatively called "truly" DE if it has a Bonferroni adjusted p-value of less than 0.05. A gene is called "truly" not DE if it has an unadjusted p-value greater than 0.05. We will then evaluate the ability of the DE methods to recover the information in the comparison of ten B6 samples with ten D2 samples by smaller comparisons of *n *B6 samples and *n *D2 samples, for *n *ranging from two to five. This is done by comparing a random set of *n *B6 and *n *D2 mouse striatum samples one hundred times and then

• generating Receiver Operator Curves (ROC, a curve describing each methods True Positive Rate as a function of its False Positive Rate for a complete range of p-value cut-offs),

• calculating partial areas under the ROC for FPR less than 0.01 and

• calculating True Positives (TP) and False Positives (FP) using a Bonferroni adjusted p-value cut-off of 0.05.

## Results and discussion

### The estimation of the common variance

We begin by examining the effect of using information from different additional sources to help explain the variances observed in the Bottomly Data. That is, assessing the impact that each of the additional datasets in Table 1 can have on estimating the pooled variances of *n *B6 vs *n *D2 mouse striatum comparisons. Thus we only consider one additional dataset at a time and do not consider how they could interact. When used to help fit the common variance surface, using information from any of the additional data sources improve the estimate of the common variance as seen in Figure 1. This is observed through all of the average *λ*'s being higher when using additional information when compared to using only the mean. *λ *is proportional to the reciprocal of the average squared error of the variance estimates, thus a larger *λ *corresponds to a better estimate of the common variance. A *λ *value of one implies that the common variance is representative of the population variance. A *λ *of zero suggests that the common variance estimate is failing to describe the observed gene sample variances.

The more relevant the information contained in the additional data source, the greater the improvement seen in the common variance estimate. As is perhaps expected either of the two striatum tissue datasets, RNA-Seq and microarray, when used to estimate the common variance produce the largest *λ*, with microarray striatum and RNA-Seq striatum only slightly out performing hippocampus. Spleen and lung both also increase *λ *highlighting that information can still be gained from unrelated tissue types, however, liver and heart barely increase *λ *at all. This can mostly be explained by the use of liver and heart resulting in the variance of one gene, transthyretin, being severely under-estimated. If this gene is excluded the *λ *generated by using liver and heart are much similar to that of spleen and lung. Including information on gene length also has the potential to improve variance information however this appears to relatively decrease as the sample size *n *increases.

Improving the accuracy of the sample variance decreases *λ *and improving the accuracy of the common variance increases *λ*. As the sample size *n *increases, *λ *decreases. This is because as *n *increases the accuracy of the gene sample variance estimates increase. As the estimation of the gene sample variances improves, the inability of the common variance to describe the gene variances becomes more clear. The converse of this is seen in Table 2. As the information content of the additional data source improves, variance estimates from D2 mice calculated with increasing sample sizes, the ability of the common variance to describe the observed gene variances, calculated from four replicates of B6 mice, also improves. *λ *is doubled by using ten replicates of D2 mouse as opposed to nothing, that is, the average squared error of the common variance is halved.

**Table 2:** Using D2 variance estimates to estimate common variance of four B6 samples.

*n *D2 samples	0	2	3	4	5	6	7	8	9	10
*λ*	0.35	0.45	0.50	0.55	0.58	0.65	0.68	0.72	0.75	0.77

The average *λ *values calculated using a random *n *D2 mouse striatum samples to estimate the variance of a random four B6 mouse striatum samples from one hundred simulations.

### The impact of moderation on inferring differential expression

The aim of the remainder of the evaluation is to assess how the use of moderation affects inference on differential gene expression. This is done by assessing the impact of moderation on both gene ranking and sensitivity. Moderation is used to both increase the sensitivity of a test, by increasing the degrees of freedom of the variance estimate, and to improve the ranking of a test, by improving the accuracy of the variance estimate.

We will start by simply comparing the t-test (T), moderated t-test (Tshrink) and a moderated t-test using additional information (Tshrink+). For the additional data source used by Tshrink+, the four striatum RNA-Seq samples [25] in Table 1 were chosen as they gave the second highest value but were not generated from the same lab as the analysis dataset (as the microarray data were).

By first considering only four vs four comparisons, the ability of moderation to improve gene ranking is illustrated in Figure 2a where a partial average ROC curve from one hundred four vs four comparisons of B6 and D2 mouse striatum is plotted for each method. This curve shows each methods TPR for a range of FPR, where a method is deemed to have ranked genes better than another at a given FPR if its TPR is higher. Here we see that Tshrink (dark blue) performs better than T (light blue) for all FPR less than 0.01. Tshrink+ (red) offers a similar improvement again on top of that of Tshrink nearly doubling the improvement of Tshrink to T.

**Figure 3:**
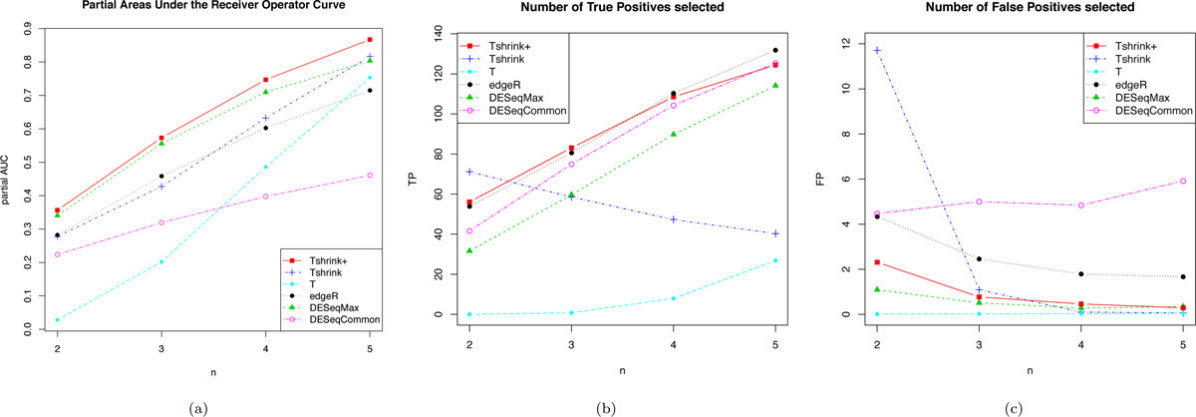
**Partial AUCs and the number of True and False Positives for a range of *n *vs *n *comparisons**. One hundred random comparisons of *n *B6 and *n *D2 mouse striatum samples a performed for six DE methods for *n *ranging from two to five. For each method and *n*, (a) partial areas under the ROC curves (partial AUC) are calculated for the regions of FPR less than 0.01 and for the conservative Bonferroni adjusted cut-off of 0.05 the average number of (b) True Positives and (c) False Positive are counted.

Moderation improves gene ranking and improving what a method moderates too can improve gene ranking further. This is again illustrated in Figure 3a, where the partial area under the ROC curve is plotted for a range of *n *vs *n *comparisons. A value of 1 corresponds to a perfect ranking and a value of zero corresponds to the most imperfect ranking. For all *n *considered Tshrink+ appears to double the improvement of Tshrink when compared to T. The relative improvements decrease as *n *increases as the information in the sample variance increases in comparison to the common variance.

Moderation can improve the sensitivity of a test for differential expression as seen in Figure 2b. Figure 2b plots the average number of True Positive genes called at varying p-value cut-offs for one hundred four vs four tests. At a Bonferroni adjusted p-value cut-off of 0.05 (the grey dashed line) T calls 8 TP, Tshrink 47 TP and Tshrink+ 108 TP. These improvements are seen at very little cost the the number of False Positives called. The number of TP and FP called at a Bonferroni adjusted p-value cut-off of 0.05 for *n *ranging from two to five are plotted in Figures 3b and 3c respectively. Here we see the number of TP called for Tshrink+ increases as *n *increases and the number FP decreasing as *n *increases. While the number of TP called also increase for T, it decreases for Tshrink over this range of *n*. The number of TP called by Tshrink will decrease until Tshrink converges to T when it will continue to increase. Tshrink may be over-zealous in its calling of TP calling a relatively large amount of FP as well for small *n*.

### Comparison with edgeR and DESeq

Tshrink+ performs favourably when compared to both DESeq and edgeR when considering gene ranking. When assessing gene ranking using Figure 3a, Tshrink+ performs marginally better than DESeqMax (green) which is better than edgeR (black) and DESeqCommon (pink). The relative performance of Tshrink+ over DESeqMax increases as *n *increases. For *n *equal to five edgeR performs worse than T. It could be argued that this is because T is becoming closer to the t-test that was used as "truth", however this behaviour is also observed when using the results from microarray array data [17] as "truth" as seen in Supplementary Figure 4 in Additional File 1. This is performance could also be explained by edgeR over moderating to a common variance that is become decreasingly relevant as *n *increases.

Tshrink+ compares comparably to edgeR and DESeq when assessing sensitivity. T selects a similar number of TP at the cut-off when compared edgeR but selects less FP as seen in Figures 3b and 3c. While DESeqMax does not select as many TP for the given cut-off as DESeqCommon it selects dramatically less FP.

## Conclusions

Using additional information improves the estimation of the common variance and the detection of differentially expressed genes. Our differential expression test, Tshrink+ which incorporates information from additional datasets, showed marked improvement in both gene ranking and sensitivity over a moderated t-test, Tshrink, and a standard t-test, T. Tshrink+ also performed favourably against edgeR and DESeq when comparing gene ranking and comparably when assessing sensitivity.

Whilst Tshrink+ can offer improvements to a differential expression analysis it also provides insight into avenues for further research. The moderation used in Tshrink+ [6] can be drastically affected by genes with unusual variances. A more sophisticated methodology which manages the influence of these genes on moderation could offer potentially large improvements. While using local regression to t the common variance when incorporating one additional dataset is easy to implement, it does not scale well to the use of multiple information sources. A parametric based approach may make the integration of multiple data sources feasible.

This methodology should be considered as a complement, not a replacement, for meta-analysis when similar studies to the RNA-Seq study of interest exist. Tshrink+ leverages only the variance estimates from external datasets to improve the variance estimation in the study of interest. If information exists on the changes of expression between conditions as well, a researcher may be remiss to not utilise this information through the use of existing meta-analysis methodologies.

Using external data to improve the estimation of the common variance for a particular problem highlights the significance of access to public data repositories like the gene expression omnibus (GEO) [26]. These repositories have the ability to actualise improved inference lending both confidence and power to results. Projects like ReCount [18] aid in this process by providing access to pre-processed data that avoids the duplication of the computationally intensive procedure of both downloading and processing large datasets.

## Competing interests

The authors declare that they have no competing interests.

## Authors' contributions

EP developed the method, implemented the algorithm and drafted the manuscript. MB, DL, and YY participated in all aspects of the study and helped to draft the manuscript. All authors read and approve of the final manuscript.

## Declarations

The publication costs for this article were funded by the corresponding author's institution.

This article has been published as part of *BMC Genomics *Volume 14 Supplement 1, 2013: Selected articles from the Eleventh Asia Pacific Bioinformatics Conference (APBC 2013): Genomics. The full contents of the supplement are available online at http://www.biomedcentral.com/bmcgenomics/supplements/14/S1.

## Supplementary Material

Additional file 1 **Additional file 1 includes a description of the normalisation used in the evaluation and additional figures**. Click here for file
