# Supplementary material for: Improved moderation for gene-wise variance estimation in RNA-Seq via the exploitation of external information
Source: BMC Genomics. 2013 Jan 21;14(Suppl 1):S9. doi: 10.1186/1471-2164-14-S1-S9 (PMC3549800; doi:10.1186/1471-2164-14-S1-S9)
Supplement: Additional file 1 — includes a description of the normalisation used in the evaluation and additional figures. [file 1471-2164-14-S1-S9-S1.docx]

Normalization Methods
GC content
In order to model the disparate library sizes and biases of PCR amplification observed in the data, a cyclic robust linear model was used. Using the first sample in the dataset as a reference, M values were calculated for each gene in the remaining samples and a straight line was fitted through the M-value vs GC-content space. The M-values were then normalised to this line.

### Other Technical effects

1. The pooled correlations of the counts for house-keeping genes are calculated.
2. Hierarchical clustering is performed using the distance 1 – correlation.
3. Tree is cut into k groups (we arbitrarily choose k equal to four).
4. For all genes, within condition residuals of the log counts are calculated.
5. LDA trained on house-keeping genes using groups from clustering, this is then used to classify all genes into groups.
6. The posterior probability of a gene belonging to a particular group is converted to a quantile value of the normal distribution.
7. Genes are then normalised to the loess curve that is fitted through the pair-wise MQ plots. (M – values vs Q – values) using the first sample as a reference.

 **Figure 1** Average TPR and FPR are calculated from 100 random four B6 vs four D2 mouse striatum comparisons for four normalisation methods using results from an Affymetrix array as truth. These are plotted against each other to form ROC curves. For any given FPR a method with a larger TPR is deemed to have ranked the genes better.

**Figure 2** Average TPR and FPR are calculated from 100 random four B6 vs four D2 mouse striatum comparisons for four normalisation methods using results from an Illumina array as truth. These are plotted against each other to form ROC curves. For any given FPR a method with a larger TPR is deemed to have ranked the genes better.

**Figure 3** Boxplots of the log variance of the within sample gene ranks for four normalisation methods. All normalisation methods on average reduce the variance of the ranking of the genes.

**Figure 4** Average TPR and FPR are calculated from 100 random four B6 vs four D2 mouse striatum comparisons and 100 random five vs five D2 mouse striatum comparisons for six DE methods. These are calculated using results from an Affymetrix array experiment as truth. The TPR and FPR are plotted against each other to form ROC curves and displayed in the region for FPR less than 0.1 as this is most relevant for calling DE. For any given FPR a method with a larger TPR is deemed to have ranked the genes better. T and Tshrink both improve in performance relative to edgeR and DESeq when moving from the four vs four comparison to the five vs five comparison.
